# Method for measurement of bacillithiol redox potential changes using the Brx-roGFP2 redox biosensor in *Staphylococcus aureus*

**DOI:** 10.1016/j.mex.2020.100900

**Published:** 2020-04-24

**Authors:** Vu Van Loi, Haike Antelmann

**Affiliations:** Freie Universität Berlin, Institute of Biology-Microbiology, D-14195 Berlin, Germany

**Keywords:** Genetically encoded roGFP2 biosensors, Microplate reader measurements, Bacillithiol, Staphylococcus aureus

## Abstract

Recent advances in the design of genetically encoded redox biosensors, such as redox-sensitive GFP (roGFP) have facilitated the real-time imaging of the intracellular redox potential in eukaryotic cells at high sensitivity and at spatiotemporal resolution. To increase the specificity of roGFP2 for the interaction with the glutathione (GSH)/ glutathione disulfide (GSSG) redox couple, roGFP2 has been fused to glutaredoxin (Grx) to construct the Grx-roGFP2 biosensor. We have previously designed the related Brx-roGFP2 redox biosensor for dynamic measurement of the bacillithiol redox potential (*E*_BSH_) in the human pathogen *Staphylococcus aureus*. Here, we describe the detailed method for measurements of the oxidation degree (OxD) of the Brx-roGFP2 biosensor in *S. aureus* using the microplate reader. In particularly, we provide details for determination of the *E*_BSH_ changes during the growth and after oxidative stress. For future biosensor applications at the single cell level, we recommend the design of genome-encoded roGFP2 biosensors enabling stable expression and fluorescence in bacteria.

• Brx-roGFP2 is specific for measurements of the bacillithiol redox potential in *Staphylococcus aureus* cells
• Control samples for fully reduced and oxidized states of Brx-roGFP2 are required for calibration during OxD measurements
• Easy to measure fluorescence excitation intensities at the 405 and 488 nm excitation maxima using microplate readers

## Graphical-abstract

**Figure:**
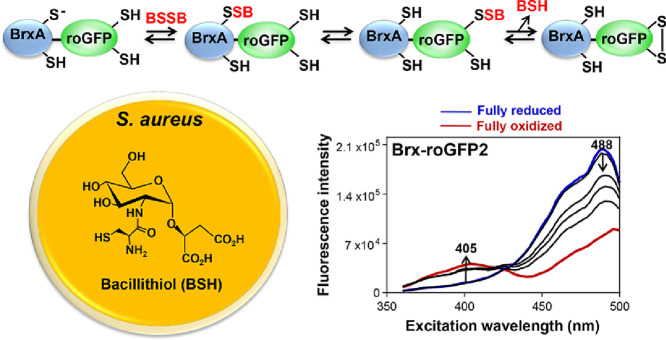

Specifications Table

**Table:** 

Subject Area	Immunology and Microbiology
More specific subject area	*Redox biology of pathogenic bacteria*
Method name	*Brx-roGFP2 biosensor measurement method*
Name and reference of original method	*Original reference for Brx-roGFP2 biosensor construction, measurements and application in* S. aureus*:* *V.V. Loi, M. Harms, M. Müller, N.T.T. Huyen, C.J. Hamilton, F. Hochgräfe, J. Pane-Farre, H. Antelmann, Real-time imaging of the bacillithiol redox potential in the human pathogen Staphylococcus aureus using a genetically encoded bacilliredoxin-fused redox biosensor, Antioxid Redox Signal 26(15) (2017) 835-848. doi: 10.1089/ars.2016.6733.*
Resource availability	*All resources including software, hardware and materials necessary to reproduce the method are described in the Methods details.*

## Method overview

*Staphylococcus aureus* is an important human pathogen that frequently encounters reactive oxygen species (ROS) by activated macrophages and neutrophils during infections. To survive under oxidative stress, *S. aureus* utilizes the low-molecular-weight (LMW) thiol bacillithiol (BSH), which serves as glutathione (GSH) surrogate to maintain the intracellular redox balance [1].

**Fig. 1:**
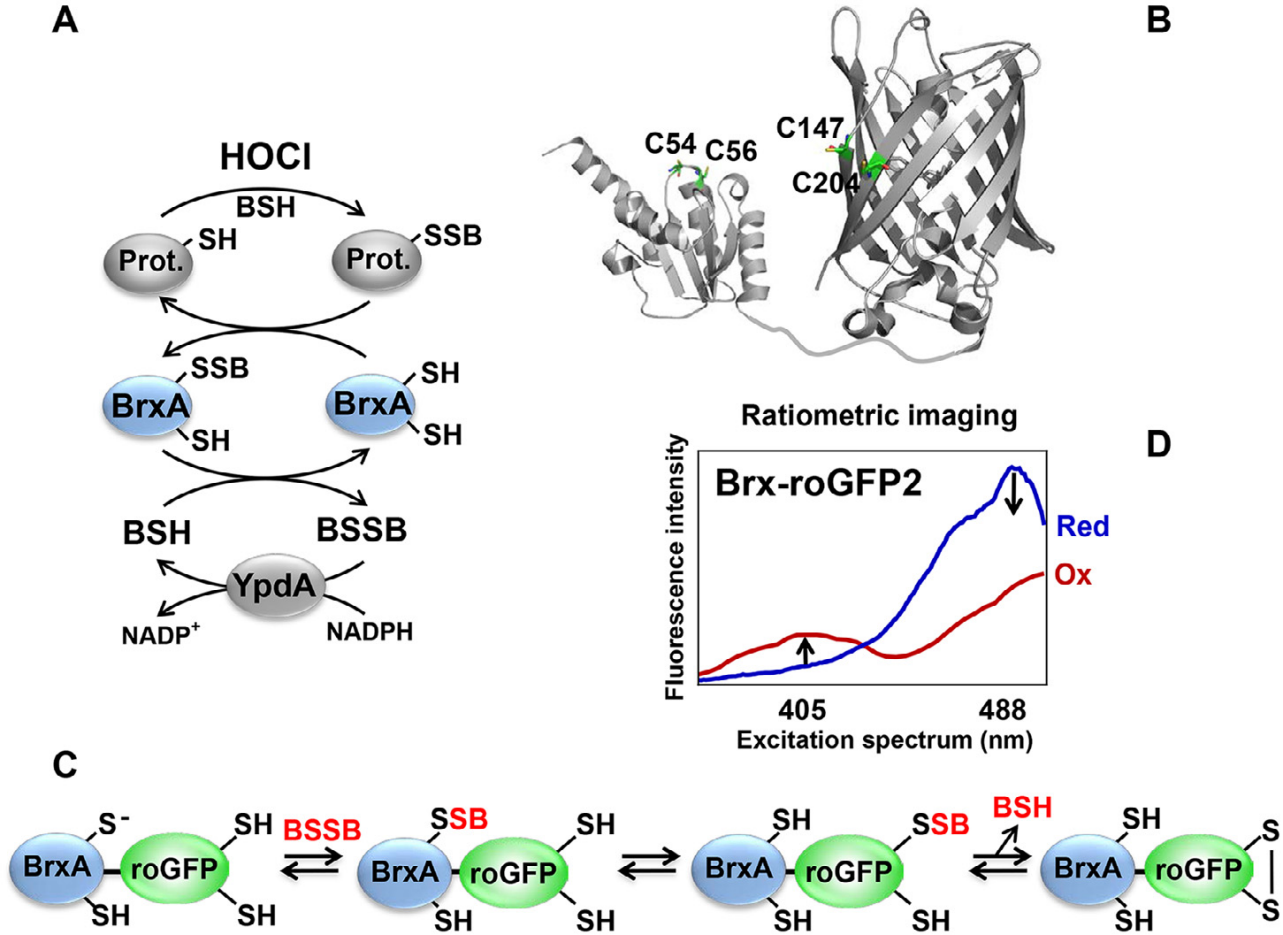
The Brx redox pathway **(A)**, structural model of the Brx-roGFP2 biosensor **(B)**, principle of Brx-roGFP2 biosensor oxidation **(C)** and ratiometric changes of the roGFP2 excitation spectrum **(D). (A)** BrxA reduces *S*-bacillithiolated proteins, resulting in Brx-SSB formation. Recycling of BrxA requires BSH and the NADPH-dependent BSSB reductase YpdA. **(C)** The Brx-roGFP2 biosensor reacts first with BSSB at the active site Cys of Brx, leading to Brx-SSB formation, subsequent transfer of the BSH moiety to the coupled roGFP2, and re-arrangement to the roGFP2 disulfide. The roGFP2 disulfide causes a change of the 405/488 nm excitation ratio. **(D)** The excitation spectrum of reduced (blue) and oxidized roGFP2 (red). roGFP2 has two excitation maxima at 405 and 488 nm. In reduced roGFP2, the 405 nm excitation maximum is low and that at 488 nm is high. Oxidation of roGFP2 leads to ratiometric changes of the 405 and 488 nm excitation maxima, resulting in an increased 405/488 ratio.

In eukaryotes, redox changes lead to oxidation of GSH to glutathione disulfide (GSSG) resulting in a decreased GSH/GSSG redox ratio and an increased GSH redox potential (*E*_GSH_). Genetically encoded redox-sensitive GFPs (roGFPs) are powerful tools to measure dynamic *E*_GSH_ changes in real-time at high sensitivity and spatiotemporal resolution in living cells and various organelles [2], [3], [4]. To increase their specificity towards the GSH/GSSG redox pair, roGFP2 probes have been fused to human glutaredoxin (Grx1). The Grx1-roGFP2 fusion allows specific equilibration between the GSH/GSSG and roGFP2_red_/ roGFP2_ox_ redox couples [3], [4], [5]. Oxidation of roGFP2 influences the spectral properties of the chromophore [4]. In reduced roGFP2, the fluorescence intensity at the 405 nm excitation maximum is low, while intensity at 488 nm excitation maximum is high. Disulfide bond formation between Cys147 and Cys204 of roGFP2 leads to ratiometric changes of the fluorescence intensities at the 405 nm and 488 nm excitation maxima [3]. The 405/488 nm excitation ratio is calculated as oxidation degree (OxD) of the biosensor which reflects the intracellular *E*_GSH_ in eukaryotic cells [3,4]. We have previously constructed a Brx-roGFP2 fused biosensor to monitor BSH redox potential (*E*_BSH_) changes during the growth, under oxidative stress and after antimicrobial treatments in the wild type and different mutant backgrounds that are impaired in redox homeostasis (Fig. 1) [6], [7], [8], [9]. The Brx-roGFP2 biosensor is highly specific for bacillithiol disulfide (BSSB) *in vitro* and responds differentially to H_2_O_2_ and HOCl *in vivo* [6]. In this work, we provide the methodological details of *E*_BSH_ measurements using the microplate reader that are related to our previous publication [6]. The applications of Brx-roGFP2 expressing cells are focused on injection assays with oxidants and OxD measurements during the growth [6]. For each sample, fully reduced and oxidized controls have to be included which are used for normalization of the OxD values. In the following sections, the detailed protocol is described regarding *S. aureus* growth, harvesting and measurements of the Brx-roGFP2 biosensor response. The method is applicable also for other bacteria to measure intrabacterial redox changes at high spatiotemporal resolution without cell disruption.

## Method details

### Materials for biosensor measurements and bacterial cultivation

1. Phosphate-buffered saline (PBS)
2. Reaction buffer: 100 mM potassium phosphate buffer with 1 mM EDTA, pH 7.0
3. Stock solutions of thiol-reactive compounds:
  • 200 mM dithiothreitol (DTT),
  • 100 mM diamide (Dia),
  • 0.02, 0.2, 1 and 2 M H_2_O_2_ (35% w/v)
  • 0.2, 0.4, 1 and 2 mM NaOCl (10-15%)
  • 1 M N-ethylmaleimide (NEM)
4. Xylose (20% w/v)
5. Chloramphenicol (Cm) stock (10 mg/ml)
6. Micro Bio-Spin^TM^ 6 columns (Biorad)
7. Growth media: Luria bertani (LB) medium and Belitsky minimal medium (BMM) [10]
8. Black flat-bottomed 96-wells plates (BD Falcon - Biosciences)
9. Microplate reader (e.g. CLARIOstar from BMG Labtech and MARS software version 3.10)

### Biosensor and control strains

1. *Staphylococcus aureus* COL expressing pRB473-*brx-roGFP2* [6]
2. *Staphylococcus aureus* COL pRB473 empty plasmid used as blank [6]

### Experimental Procedures

#### (A) Measurements of Brx-roGFP2 biosensor responses in S. aureus during the growth in vivo

The Brx-roGFP2 biosensor was cloned under control of the xylose-inducible promoter P_XylR_ into plasmid pRB473, which was transduced into *S. aureus* COL wild type and various mutant strains [6,7]. For measurements of the Brx-roGFP2 biosensor response during the growth, *S. aureus* COL pRB473-brx-roGFP2 was cultivated in LB medium with 1% xylose and cells harvested during different times along the growth (exponential growth phase, transition to stationary phase and later stationary phase) [6]. The methods details for bacterial growth, harvesting and measurements are as follows:

*Preparation of S. aureus cells expressing the Brx-roGFP2 biosensor*

1. Growth of the overnight cultures of *S. aureus* COL-pRB473-*brx-roGFP2* (biosensor strain) and *S. aureus* COL-pRB473 (control strain with empty plasmid used as blank) in 20 ml LB medium with 1 % xylose and 10 µg/ml Cm in a shaking water bath at 37°C for 16 h.
2. Inoculation of the overnight cultures into fresh LB medium with 1 % xylose and 10 µg/ml Cm to an optical density at 540 nm (OD_540_) of 0.1. Cultivation of *S. aureus* strains under vigorous agitation at 37°C for sampling at different time points along the growth.
3. Sampling of 3 × 10 ml each at the 3, 4, 5 and 6 hour time points after inoculation.
  • One sample is treated with 10 mM NEM to block free thiols of Brx-roGFP2 expressed in *S. aureus* cells. NEM alkylation is required to avoid changes in biosensor oxidation in *S. aureus* cells during sample processing, which can also lead to stress exposure in *S. aureus*. NEM is a membrane-permeable thiol-trapping reagent, which rapidly alkylates accessible thiols in intact cells *in vivo* and in protein extracts after cell lysis [11], [12], [13], [14]. Concentrations of 10-20 mM NEM were previously used to freeze successfully the redox state of roGFP2 biosensors expressed in HeLa cells and *Plasmodium falciparum in vivo* [2,15]. Thus, we applied 10 mM NEM to block free thiols of Brx-roGFP2 inside *S. aureus* cells before sample harvesting and fluorescence measurements.
  • The other two samples are used as fully oxidized and reduced controls and treated with 5 mM Dia and 10 mM DTT, respectively, followed by NEM alkylation. The alkylated sample and controls are harvested by centrifugation at 8500 rpm for 10 min at 25°C. *Note:* High fluorescence signals of biosensor cells at both 405 and 488 nm wavelengths are required for ratiometric quantification of the Brx-roGFP2 response. Thus, harvest higher volumes of e.g. 20-30 ml cultures at lower OD values [6].
4. Washing and resuspension of *S. aureus* cells in 500 µl PBS with 50 mM NEM to concentrate the cells 20-fold for enhanced fluorescence signals. The samples are ready for measurements of Brx-roGFP2 fluorescence intensities using the microplate reader and confocal imaging.

*Preparation of the microplates with S. aureus cells for measurements using microplate reader*

1. For each time point, transfer 200 µl of concentrated cells of *S. aureus* COL-pRB473-*brx-roGFP2* (biosensor strain) and COL-pRB473 (strain with empty plasmid) including fully reduced and oxidized controls into each well of the black flat-bottomed 96-wells microplates
  • Blank (B): 200 µl *S. aureus* COL pRB473
  • Fully reduced control: 200 µl *S. aureus* COL pRB473-*brx-roGFP2* (DTT-treated)
  • Fully oxidized control: 200 µl *S. aureus* COL pRB473-*brx-roGFP2* (Dia-treated)
  • Sample X: 200 µl *S. aureus* COL pRB473-*brx-roGFP2* at time point X
2. Centrifuge the microplate (e.g. with Heraeus Multifuge 3XR centrifuge, Thermo Scientific) shortly for 5 min at 800 rpm at 25°C. Check if cells are equally distributed at the bottom of the microplate wells and start measurements.

*Setup the microplate reader for measurements of Brx-roGFP2 fluorescence*

Measurements of roGFP2 fluorescence can be performed using various microplate readers that are equipped with 405 and 488 nm excitation filters and a 510 nm emission filter. Due to recommendation by various roGFP2 users, we applied the CLARIOstar microplate reader and the related software version 5.20 R5. Here we provide the comprehensive protocol of previous Brx-roGFP2 measurements. Thus, the description is related to the CLARIOstar reader used in our lab to generate previous results [6]. First, the temperature is set to 37°C and the new measurement protocol is created under “Test Protocols” based on the following parameters:

1. Select “Method”: Fluorescence Intensity
2. Select “Measurement mode”: Plate mode
3. Select “Basic Parameters”:
  • Microplate: FALCON 96, Focal height 4,5 mm
  • *Optic Setting*: No. of multichromatics 2, Presets Fluorescein (FTIC), Excitation 405 nm, bandwidth 10 (405-10), Dichroic: 457.5, Emission 510 nm, bandwidth 10 (510-10)
  • *Orbital Averaging*: On, Diameter 3 mm
  • Optic: Bottom optic
  • *General settings*: Settling time 0.5, Number of kinetic windows 1
  • *Kinetic Windows 1*: No. of cycles 10, No. of flashes per well and cycle 10
4. Select “Concentrations/Volumes/Shaking”:
  • *Standard Concentrations*: Factor 1
  • *Shaking Options*: Shaking mode Double orbital, Frequency 300 rpm, Time 10 sec, Shake before each cycle, Idle movement None
5. Select Multichromatic:
  • Number 1: Fluorescein (FTIC), Excitation 405 nm, bandwidth 10 (405-10), Dichroic 457.5, Emission 510 nm, bandwidth 10 (510-10)
  • Number 2: Fluorescein (FTIC), Excitation 488 nm, bandwidth 10 (488-10), Dichroic 499, Emission 510 nm, bandwidth 10 (510-10)
6. Press “Start measurements” and select “Focus and Gain Adjustment/Plate ID” option to adjust Gain of both excitations wavelengths of fully reduced and oxidized controls
  • *Layout*: Select microplate well with fully oxidized Dia control
  • *Focus and Gain adjustment*: monochromator/ filter setting 1: 405-10/510-10, Selected well (Dia control), Target value 95 %, Start adjustment
  • *Layout*: Select microplate well with fully reduced DTT control
  • *Focus and Gain adjustment*: monochromator/ filter setting 2: 488-10/510-10, Selected well (DTT control), Target value 95 %, Start adjustment
  • Press “Start measurement” to measure fluorescence intensities of your samples X
7. Export and analyze the data using the software MARS version 3.10.
8. Data analysis: *S. aureus* cells with empty pRB473 plasmid are used as blank (B). Fluorescence emission intensities are measured for fully oxidized (Dia) and fully reduced (DTT) controls and the samples after excitation at both 405 and 488 nm. All fluorescence intensities of controls and samples are blank subtracted and further analyzed by the MARS 3.10 software.
9. The OxD of the Brx-roGFP2 biosensor is determined for each sample and normalized to fully reduced and oxidized controls as described using equation (1) [6].
  (1) $$\text{OxD}=\frac{I405_{\text{sample}\;}\times \;I488_{\text{red}\;}-\;I405_{\text{red}\;}\times \;I488_{\text{sample}}}{I405_{\text{sample}\;}\times \;I488_{\text{red}\;}-\;I405_{\text{sample}\;}\times \;I488_{\text{ox}}\;+\;I405_{\text{ox}\;}\times \;I488_{\text{sample}}-\;I405_{\text{red}\;}\times I488_{\text{sample}}}\;$$
  • *I*405 and *I*488 are observed fluorescence excitation intensities at 405 nm and 488 nm of the samples, respectively.
  • *I*405red, *I*488red, *I*405ox and *I*488ox are fluorescence excitation intensities at 405 nm and 488 nm of fully reduced and oxidized controls, respectively.
10. Based on the OxD values and the previously determined $E_{\text{roGFP}2}^{o^{\prime }}$ = - 280 mV [16], the BSH redox potential (*E*_BSH_) can be calculated using to the Nernst equation (2):
  (2) $$E_{\text{BSH}}=E_{\text{roGFP}2}=E_{\text{roGFP}2}^{o{}^{\prime }}-(\frac{\text{RT}}{2F})*\text{In}(\frac{1-\text{OxD}}{\text{OxD}})$$

#### (B) Measurements of Brx-roGFP2 responses in S. aureus after exposure to oxidants in vivo

For measurements of the Brx-roGFP2 biosensor response after exposure to oxidants, *S. aureus* COL pRB473-*brx-roGFP2* cells are harvested from the LB overnight culture. Cells are transferred to minimal medium and filled into the microplate wells. Oxidants are directly injected into the microplate wells with the biosensor cells. The methods details are as follows:

*Preparation of S. aureus cells expressing the Brx-roGFP2 biosensor for injection assays.*

1. Growth of the overnight cultures of *S. aureus* COL-pRB473-*brx-roGFP2* (biosensor strain) and COL-pRB473 (strain with empty plasmid used as blank) in 20 ml LB medium with 1 % xylose and 10 µg/ml Cm in a shaking water bath at 37°C for 16 h.
2. Sampling of 20 ml cells by centrifugation at 8500 rpm at 25°C.
3. Washing and dilution of cells in BMM with 1% xylose and 10 µg/ml Cm, adjustment to an OD_500_ of 2 to start injection assays.

*Preparation and measurements of the microplates with S. aureus cells in oxidant injection assays*

1. Transfer of 190 µl of *S. aureus* COL-pRB473-brx-roGFP2 (biosensor strain) and *S. aureus* COL-pRB473 (strain with empty plasmid used as blank) with an OD500 of 2 to the microplate wells
  • Blank (B): 190 µl *S. aureus* COL pRB473 + 10 µl PBS puffer
  • Fully reduced control: 190 µl *S. aureus* COL pRB473-brx-roGFP2 + 10 µl 200 mM DTT
  • Fully oxidized control: 190 µl *S. aureus* COL pRB473-brx-roGFP2 + 10 µl 100 mM Dia
  • Note: Mix controls well with DTT and Dia and avoid air bubbles.
  • Sample X: 190 µl *S. aureus* COL pRB473-brx-roGFP2 + 10 µl oxidant
  • Note: Injection of 10 µl oxidants (H_2_O_2_ and NaOCl) is performed directly into the microplate wells before the start of the microplate measurements. Since *S. aureus* is resistant to H_2_O_2_, we used 10 µl of 20-fold H_2_O_2_ stock solutions of 0.02, 0.2, 1 and 2 M H_2_O_2_ resulting in final doses of 1, 10, 50 and 100 mM H_2_O_2_, respectively, to see the increased biosensor response [6]. In contrast to H_2_O_2_, *S. aureus* is very sensitive to NaOCl and the biosensor fully oxidized by 50-100 µM NaOCl [6]. Thus, we used 10 µl of 20-fold NaOCl stock solutions of 0.2, 0.4, 1 and 2 mM NaOCl, corresponding to final concentrations of 10, 20, 50 and 100 µM NaOCl, respectively, to reveal increased oxidation and recovery of Brx-roGFP2 inside *S. aureus* cells [6].
2. Incubate the microplate in the microplate reader for 10 min to ensure complete reduction and oxidation of the DTT and DIA-treated controls, respectively.
3. Setup the microplate reader as described in A).
4. Start measurement and pause after the 20th cycle. Add 10 µl of the oxidants (NaOCl or H_2_O_2_) directly into the wells to the 190 µl samples and continue Brx-roGFP2 measurement immediately.
5. Export and analyze the data using the software MARS version 3.10 as described in A).

#### (C) Measurements of Brx-roGFP2 biosensor responses by BSSB in vitro

**Fig. 2:**
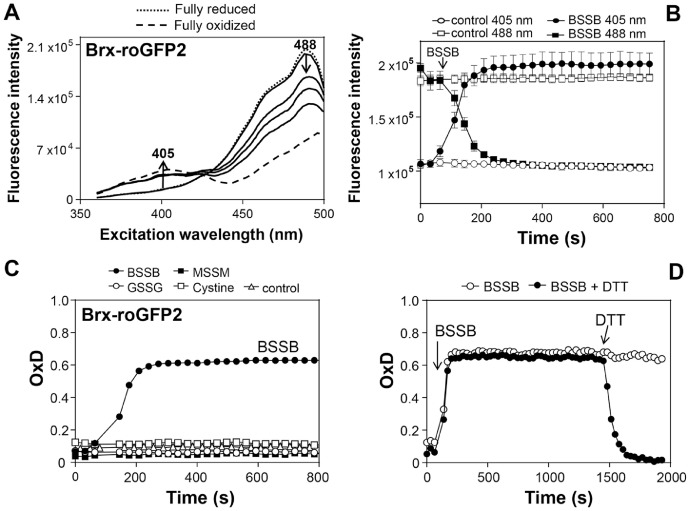
**Ratiometric response of the purified Brx-roGFP2 biosensor to BSSB *in vitro*. (A)** Purified Brx-roGFP2 protein was treated with 100 µM BSSB for 60, 120 and 240 sec and alkylated with 10 mM NEM. The fluorescence excitation spectra of Brx-roGFP2 were scanned using the microplate reader. **(B)** The purified Brx-roGFP2 biosensor was treated with 100 µM BSSB and the ratiometric changes of the fluorescence intensities at the 405 and 488 nm excitation maxima were measured using the microplate reader. Upon oxidation, the 405nm excitation maximum increases while the 488nm excitation maximum decreases which is shown for both wavelengths separately. **(C)** Brx-roGFP2 responds specifically to BSSB, but not to other LMW thiol disulfides (GSSG, MSSM, cystine). The Brx-roGFP2 biosensor was treated with 50 µM of the LMW thiol disulfides. **(D)** Purified Brx-roGFP2 reacts fast and reversible with BSSB as shown by treatment with 50 µM BSSB for 20 min and subsequent reduction with 10 mM DTT.

The specific response of purified Brx-roGFP2 protein is analyzed after exposure to BSSB in comparison to other LMW thiol disulfides *in vitro* (e.g. cystine, GSSG, MSSM) [6]. The concentrations of the LMW thiol disulfides are in the physiological range as determined in previous studies using monobromobimane derivatisation of LMW thiols [17]. The methods details are as follows:

1. Prepare stock solutions for biosensor reduction and oxidation including 100 mM DTT, 50 mM Dia and 10-fold stock of LMW thiol disulfides (BSSB, GSSG, cystine) on ice.
2. Reduce purified Brx-roGFP2 protein with 10 mM DTT for 20 min on ice, equilibrate Micro Bio-Spin^TM^ 6 columns with 500 µl reaction buffer and load 75 µl of reduced Brx-roGFP2 to the column to remove excess DTT. Process protein sample as fast as possible to avoid air-oxidation.
3. Measure Brx-roGFP2 concentration by Nanodrop2000 (Thermofisher) and calculate concentration in µM based on the molecular weight (MW) and extinction coefficients. (http://web.expasy.org/protparam/). The amino acid sequence of Brx-roGFP2 is available in Fig. S1.
4. Prepare the microplate plate for measurements. Dilute purified Brx-roGFP2 into reaction buffer to 1µM. Add 90 µl of 1 µM purified Brx-roGFP2 protein into each well:
  • Blank: 100 µl reaction buffer
  • Control for baseline: 90 µl Brx-roGFP2 + 10 µl reaction buffer
  • Fully reduced control: 90 µl Brx-roGFP2 + 10 µl 100 mM DTT
  • Fully oxidized control: 90 µl Brx-roGFP2 + 10 µl of 50 mM Dia
  • Sample X: 90 µl Brx-roGFP2 + 10 µl of 0.5 mM LMW thiol disulfides (e.g. BSSB, GSSG or cystine) (*Note*: Injection of LMW thiol disulfides is performed directly into the microplate wells shortly before the start of measurements as described in B)
5. Prepare the microplate reader for measurements using the instrument setup parameters as described in A) with modifications: Temperature setting 25°C, settling time 0.2. *Note*: The temperature is set to 25°C to avoid air-oxidation of the purified Brx-roGFP2 biosensor. For scanning the fluorescence excitation spectrum, select the measurement mode “Spectral scanning”, the scanning range from 360 to 500 nm and the bandwidth of 10 nm using 510 nm as emission filter. Example results for injection assays after BSSB treatment of purified Brx-roGFP2 biosensor are shown in Fig. 2 and in the previous publication [6].

## Declaration of Competing Interest

The authors declare that they have no known competing financial interests or personal relationships that could have appeared to influence the work reported in this paper.

## Appendix Supplementary materials

Image, application 1
