# Supplementary material for: Method for measurement of bacillithiol redox potential changes using the Brx-roGFP2 redox biosensor in Staphylococcus aureus
Source: MethodsX. 2020 Apr 24;7:100900. doi: 10.1016/j.mex.2020.100900 (PMC7214941; doi:10.1016/j.mex.2020.100900)
Supplement: Supplementary file 1 [file mmc1.pdf]

Figure S1: The protein sequence of the BrxA-roGFP2 biosensor

MNAYDAYMKEIAQQMRGELTQNGFTSLETSEAVSEYMNQVNADDTTFVVINSTCGCAAG
LARPAAVAVATQNEHRPTNTVTVFAGQDKEATATMREFIQQAPSSPSYALFKGQDLVYFMP
REFIEGRDINDIAMDLKDAFDENCKTSGGSGGGGSGGGGSGGGGSGGGGSGGGGSGGE
FVSKGEELFTGVVPILVELDGDVNGHKFSVSGEGEGDATYGKLTLKFISTTGKLPVPWPTLVT
TLTYGVQCFSRYPDHMKQHDFFKSAMPEGYVQERTIFFKDDGNYKTRAEVKFEGDTLVNRI
ELKGIDFKEDGNILGHKLEYNYNCHNVYIMADKQKNGIKVNFKIRHNIEDGSVQLADHYQQ
NTPIGDGPVLLPDNHYLSTCSALSKDPNEKRDHMVLLEFVTAAGITLGMDELYKHHHHHH
